# Patterns of Evolutionary Conservation of Essential Genes Correlate with Their Compensability

**DOI:** 10.1371/journal.pgen.1002803

**Published:** 2012-06-28

**Authors:** Tobias Bergmiller, Martin Ackermann, Olin K. Silander

**Affiliations:** 1 Department of Environmental Systems Science, ETH Zurich, Zurich, Switzerland; 2 Department of Environmental Microbiology, Eawag, Dubendorf, Switzerland; 3 Computational and Systems Biology, Biozentrum, University of Basel, Basel, Switzerland; Université Paris Descartes, INSERM U1001, France

## Abstract

Essential genes code for fundamental cellular functions required for the viability of an organism. For this reason, essential genes are often highly conserved across organisms. However, this is not always the case: orthologues of genes that are essential in one organism are sometimes not essential in other organisms or are absent from their genomes. This suggests that, in the course of evolution, essential genes can be rendered nonessential. How can a gene become non-essential? Here we used genetic manipulation to deplete the products of 26 different essential genes in *Escherichia coli*. This depletion results in a lethal phenotype, which could often be rescued by the overexpression of a non-homologous, non-essential gene, most likely through replacement of the essential function. We also show that, in a smaller number of cases, the essential genes can be fully deleted from the genome, suggesting that complete functional replacement is possible. Finally, we show that essential genes whose function can be replaced in the laboratory are more likely to be non-essential or not present in other taxa. These results are consistent with the notion that patterns of evolutionary conservation of essential genes are influenced by their compensability—that is, by how easily they can be functionally replaced, for example through increased expression of other genes.

## Author Summary

In any given organism, a fraction of all genes in the genome are required for viability; if they are experimentally deleted, the organism dies. Interestingly, the set of essential genes is usually not identical even for closely related organisms. Genes that are essential in one organism are sometimes nonessential in sister taxa or even missing from their genomes. This suggests that, in the course of evolution, some genes can be rendered non-essential and consequently can be lost. How can genes become non-essential? It is possible that changes in an organism's living conditions render previously essential functions unessential. Alternatively, it is possible that, during evolution, the function of an essential gene can be taken over by another gene, so that the essential gene becomes dispensable. Here, we tested the second hypothesis experimentally in the laboratory. We tried to replace the functions of essential genes in the bacterium *Escherichia coli*. We find that the genes that can easily be replaced in the laboratory are also more likely to be lost in the course of evolution. This suggests that differences in the evolutionary fate between essential genes can be partially explained by how easily their functions can be taken over by other genes.

## Introduction

**Figure 1:**
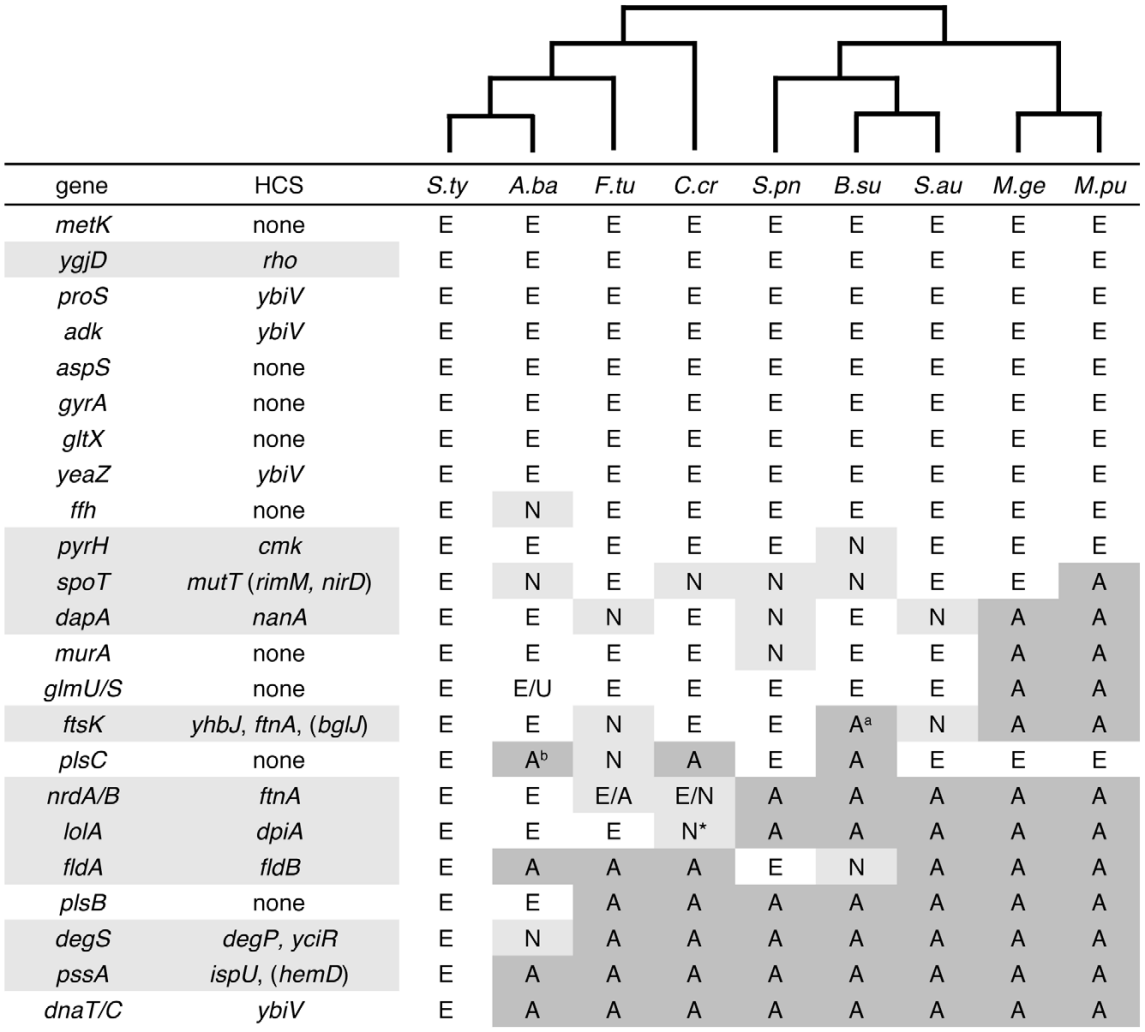
Few high copy suppressors are found for conserved and consistently essential genes. We gathered information on orthologous genes in nine additional taxa for which essentiality has been experimentally investigated. All genes listed are essential in *E.* coli; for each gene, the orthologue in other taxa is indicated as essential (E; white background), non-essential (N; light grey), associated with large fitness reduction (N*), or unknown (U). Cases in which there is no orthologue are indicated with an A (absent; dark grey background). For example, *spoT*, which is essential in *E. coli*, has been found to be essential in only four out of eight other taxa and is absent from one. The second column indicates the high copy suppressors that were isolated (genes for which suppressors were isolated are highlighted in light grey). In parentheses are high copy suppressors that were recovered from the screen but which were not analyzed further. A cladogram showing the evolutionary relationships of these taxa is shown at the top of the table. Abbreviations: *S.ty, Salmonella typhi Ty2; A.ba, Acinetobacter baylyi; C.cr, Caulobacter crescentus NA100; F.tu, Francisella tularensis U112; S.pn, Streptococcus pneumonia TIGR4; B.su, Bacillus subtilis 168; S.au, Staphylococcus aureus 8325; M.ge, Mycoplasma genitalium G37; M.pu, Mycoplasma pulmonis UAB CTIP*. a) No reciprocal best hit orthologue of *ftsK* exists in *B. subtilis* due to an apparent duplication (genes BSU16800 and BSU29805); neither gene is essential. b) No reciprocal best hit orthologue of *plsC* exists in *F. tularensis* due to an apparent duplication (FTN1749 and FTN1750); only FTN1749 is essential.

Essential genes code for central cellular processes required for the viability of an organism. Many recent studies have used experimental data to determine gene essentiality in a large number of bacteria [1]–[11]. The central role that essential genes play suggests they should be highly conserved during evolution, and several comparative genomic analyses have confirmed this hypothesis [12]–[15]. A second implication of this pattern of conservation is that essential genes tend to remain essential during evolution: if a gene is essential in one organism, then orthologues of that gene are usually essential in other organisms (Figure 1).

However, there are many genes that do not follow these patterns: some genes that are essential in one organism are non-essential in other organisms; in other cases, genes that are essential in one organism are absent or have been lost from the genomes of other organisms [13], [15], [16]. Instances in which essential genes have become non-essential, or have been lost completely from genomes, suggest that either changes in physiological or environmental conditions have altered the essentiality of a gene, or that the genetic context has changed in a way to allow loss of a previously essential function. In this case, a second gene (either paralogous or unrelated to the original essential gene) may now perform the essential function. This raises the question of whether there is a connection between compensability and conservation level of essential genes.

The possibility of a connection between compensability and gene conservation has been raised on at least one occasion previously. Geissler et al. [17] observed that the *Escherichia coli* cell division protein ZipA is poorly conserved in other taxa. Under the assumption that other proteins must fulfill this role in these other taxa, they looked for suppressor mutations that would obviate the requirement for ZipA, and found that a single mutation in FtsA suppressed the lethal *zipA* phenotype.

Here we used a systematic approach to investigate how frequently the functions of essential genes of *Escherichia coli* can be replaced under laboratory conditions, and whether the frequency of this process correlates with patterns of evolutionary conservation.

To gain insight into this question, we used the following methodology. First, we compromised the function of an essential gene in *Escherichia* coli by decreasing its expression with a tightly regulated promoter. We then increased the expression level of a large number of other genes to identify genes that are capable of compensating for the function of the impaired essential gene. Repeating this process for a large number of essential genes, we isolated a set of genes that can functionally replace the essential genes when overexpressed. We find that although the majority of these compensating genes are not homologous to the impaired essential genes, they exhibit similar functions. In a few cases, the compensating genes are capable of fully replacing the functions of the essential gene, allowing the deletion of the essential gene from the genome. Finally, we show that those essential genes whose function can be compensated for in the laboratory are more likely to be non-essential or not present in other bacterial genomes, raising the possibility that similar compensatory mechanisms may allow essential gene loss to occur in natural populations.

Many previous studies have shown that gene essentiality is a mutable characteristic and is dependent on both the genetic background of the organism or the environmental conditions [18]–[21] (such genes are termed conditionally essential genes). The results we present here imply that in some cases it may be possible to predict which essential genes are more likely to be conditionally essential.

## Results/Discussion

### Construction of Strains with Conditional Expression of Essential Genes

**Figure 2:**
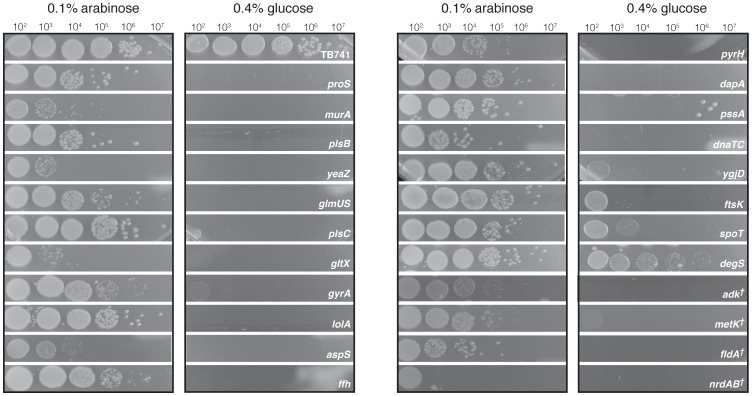
Differential colony formation of conditional lethal mutant strains on arabinose or glucose-containing LB agar plates. The native promoters of 23 genes or operon pairs were replaced with the arabinose-inducible *araBAD* promoter (see Methods). By shifting these mutant strains from medium with 0.1% L-arabinose to medium without L-arabinose and supplemented with 0.4% D-glucose, expression of the essential gene was repressed. In all cases this resulted in growth inhibition or severe growth defects. The gene whose native promoter was replaced is indicated on the right hand side of each row; the ancestral strain TB741 is shown at the top. In the case of *degS*, substantial growth occurs even after repression, suggesting that growth only becomes inhibited once the gene products are sufficiently depleted; this has been observed previously [19]. This is also true, although to a lesser extent, for *spoT*, *ftsK*, and *ygjD*. To grow the spot plates, cultures were grown overnight in 0.1% arabinose, diluted into LB medium, and 5 µl of the indicated dilution were spotted onto plates and incubated at 37° for 10 hours. Those strains indicated with a cross were incubated for 24 hours.

We constructed a collection of *Escherichia coli* strains in which essential genes were placed under the control of a conditionally expressed promoter. The essential genes were selected from a variety of functional classes [22], and exhibit a wide range of conservation levels [15]. In addition, some of these genes have been consistently found to be essential across all bacteria that have been examined empirically, while others are essential in only a few (Figure 1). A total of 26 genes were chosen (approximately 10% of the essential gene content of *E. coli*); six of these genes are in essential tandem operons (*nrdAB*, *dnaTC* and *glmUS*). To control the expression of the essential genes, we replaced their native promoters with the arabinose-inducible *araBAD* promoter (P_ara_; see Methods). By shifting these mutant strains from medium with L-arabinose to medium without L-arabinose and supplemented with D-glucose, expression of the essential gene was repressed, and in all cases this resulted in growth inhibition or severe growth defects (Figure 2). We also tested if a plasmid encoding the corresponding essential gene rescued the lethal growth phenotype, and this was the case, except for the operons *dnaTC* and *nrdAB* (Table S1). This is most likely due to the fact that these are tandem operons, and transformation and maintenance of two separate plasmids that complement the function of each gene is unlikely, as the plasmids share the same replication origins and resistance markers.

### Screening of an Overexpression Library of All *E. coli* Open Reading Frames for High Copy Suppressors

Next, we assembled a library of overexpression plasmids using the ASKA(-) strain collection [23]. The ASKA(-) library consists of 4123 clones that each contain a plasmid with one *E. coli* open reading frame cloned behind an IPTG-inducible P_lac_ promoter and a N-terminal (His)_6_-tag. Addition of IPTG induces strong expression of the downstream open reading frame. We removed the 26 clones containing plasmids with the essential genes mentioned above from the ASKA library. We then pooled the remaining 4097 clones and extracted plasmid DNA from this mixed pool.

Each conditional lethal mutant was transformed with an aliquot of the purified plasmid pool and plated on restrictive agar plates (where the essential genes were not expressed) with IPTG to induce expression of plasmid-encoded genes. We induced expression of the plasmid encoded genes with 50 µM IPTG, because higher induction levels are known to be deleterious for growth: 51% of *E. coli* proteins expressed under control of P_lac_ at 1 mM IPTG cause lethality [23]. We also measured transformation efficiency: when transformed and plated under permissive conditions with selection for the plasmid-encoded chloramphenicol resistance, all strains except *murA* and *fldA* gave rise to at least 1.2×10^4^ colonies, which is the minimal number of clones required for coverage of 95% of all variants transformed [24] (see Table S1).

We recovered up to 10 transformants from each plate, and restreaked them onto restrictive agar plates with IPTG to confirm growth. Upon successful growth of these clones, plasmids were extracted. To discriminate between possible chromosomal suppressor mutations and high copy suppressors (HCS) encoded on plasmids, each plasmid was retransformed into the ancestral conditional lethal mutant under permissive conditions, and colonies were tested for growth under restrictive conditions. In case of successful growth, the plasmid was sequenced. To control for suppression by multiple plasmids, we purified plasmids from the ASKA library and repeated the retransformation test, yielding the same results as before. For cases in which high copy suppression by the purified plasmid could not be confirmed, a second round of transformation and selection as described above was carried out.

For six strains (P_ara_-*aspS*, *plsC*, *plsB*, *ffh*, *glmUS*, and *gltX*), although colonies were recovered, no HCS plasmids were isolated after two rounds of transformation and selection (Figure 1). In these cases it is possible that chromosomal mutations were responsible for rescuing the conditional lethal phenotypes, possibly by mutation of the P_ara_ promoter to mitigate repression. In three cases, no colonies were recovered at all (P_ara_-*gyrA*, *metK*, *murA*). For *murA*, this might have been due to poor transformation efficiency. Finally, four conditional lethal mutants (P_ara_-*adk*, *dnaTC*, *proS*, *yeaZ*) were recovered repeatedly with a plasmid coding for the gene *ybiV*. We presumed that *ybiV* interfered with the function of the arabinose promoter: all five essential genes are functionally different, and expression of *ybiV* promoted growth of these mutants under all restrictive conditions. Furthermore, *ybiV* has been found previously in screens using a P_ara_ construct [25]. We thus excluded the HCS *ybiV* from the subsequent analyses.

This left us with ten essential genes for which we had identified one or more HCS (Figure 1). For each of these ten genes, we subjectively chose one HCS for further analysis – with the exception of the essential gene *degS*, for which we included both HCS.

### Complementation of Essential Genes by High Copy Suppressors

**Figure 3:**
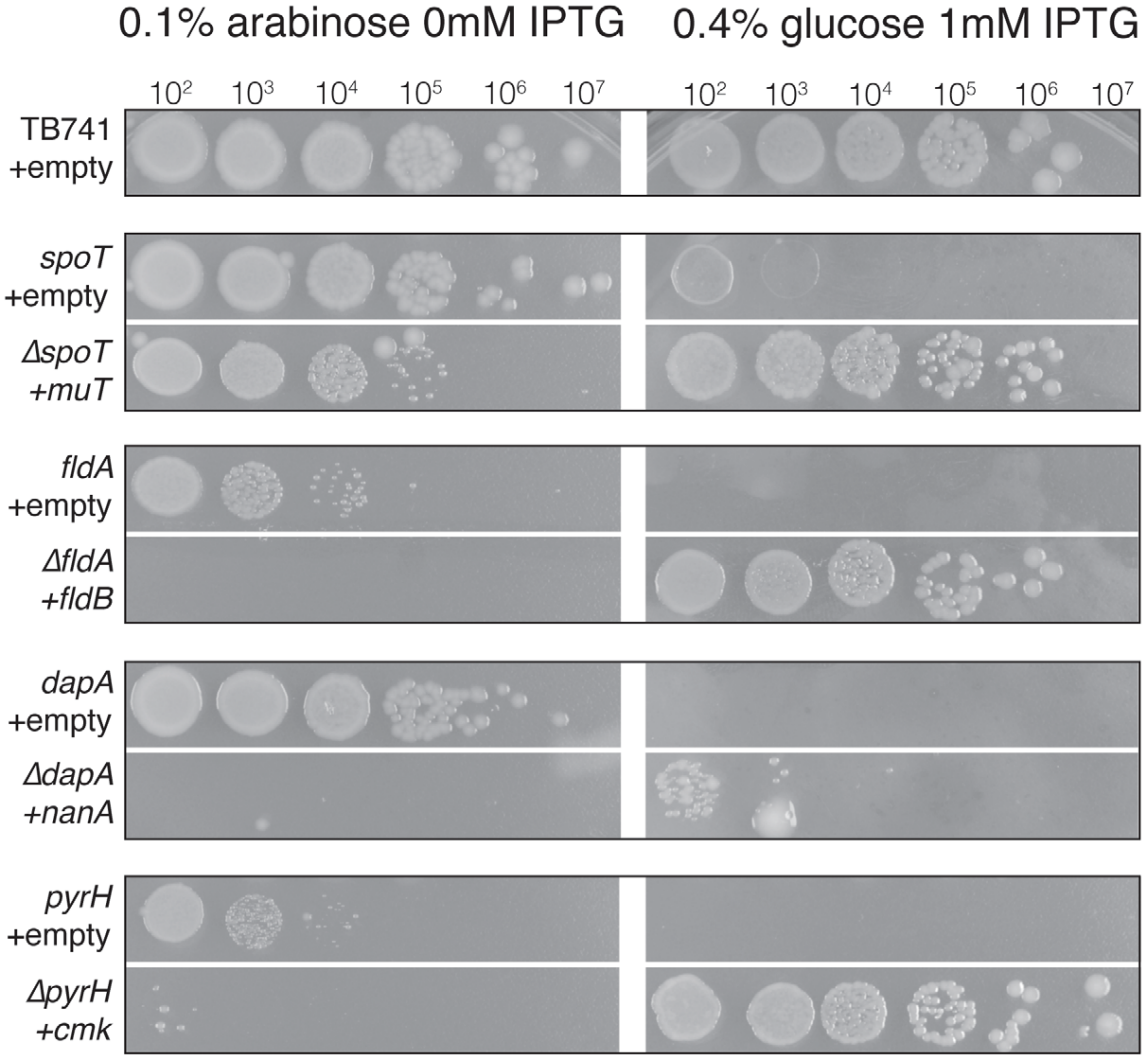
Differential colony formation of essential gene knockout mutant strains. We attempted to knock out the essential genes in strains for which we isolated an HCS. We were successful in the four cases shown here. For each essential gene, the top row illustrates the growth that is observed when the native promoter is replaced by P_ara_, under permissive (0.1% arabinose) or restrictive (0.4% glucose) growth conditions. The bottom row indicates the growth that is observed when the essential gene is knocked out, under conditions in which the suppressor is not induced (0 mM IPTG) or induced (1 mM IPTG). In the absence of the HCS, almost no growth is observed under restrictive conditions. However, when the HCS is induced, robust growth is almost always observed, even in the cases in which the essential gene is knocked out. Note that the Δ*spoT* strain is complemented by the *mutT* plasmid without induction of *mutT* expression. To grow the spot plates, cultures were grown overnight in 0.1% arabinose with 15 µg/ml chloramphenicol and 1 mM IPTG, diluted into LB medium, and 5 µl of the indicated dilution were spotted onto plates containing 0.1% arabinose with 15 µg/ml chloramphenicol, or 0.4% glucose with 1 mM IPTG. Arabinose plates were incubated for 14 hours at 37°, while glucose plates were incubated for 40 hours.

Next, we tested whether the recovered HCS plasmids could replace the functions of their respective essential genes, or whether viability might rely on low-level transcription from the repressed P_ara_ promoter. We attempted to knock out the corresponding essential genes in strains harboring the HCS plasmid, with expression of the suppressor induced using 50 µM IPTG. We were successful in four cases (these essential gene - HCS pairs were: *dapA*/*nanA*, *spoT/mutT*, *pyrH*/*cmk* and *fldA/fldB*; Figure 3). We were not able to delete these four essential genes from a strain carrying an empty control plasmid.

**Figure 4:**
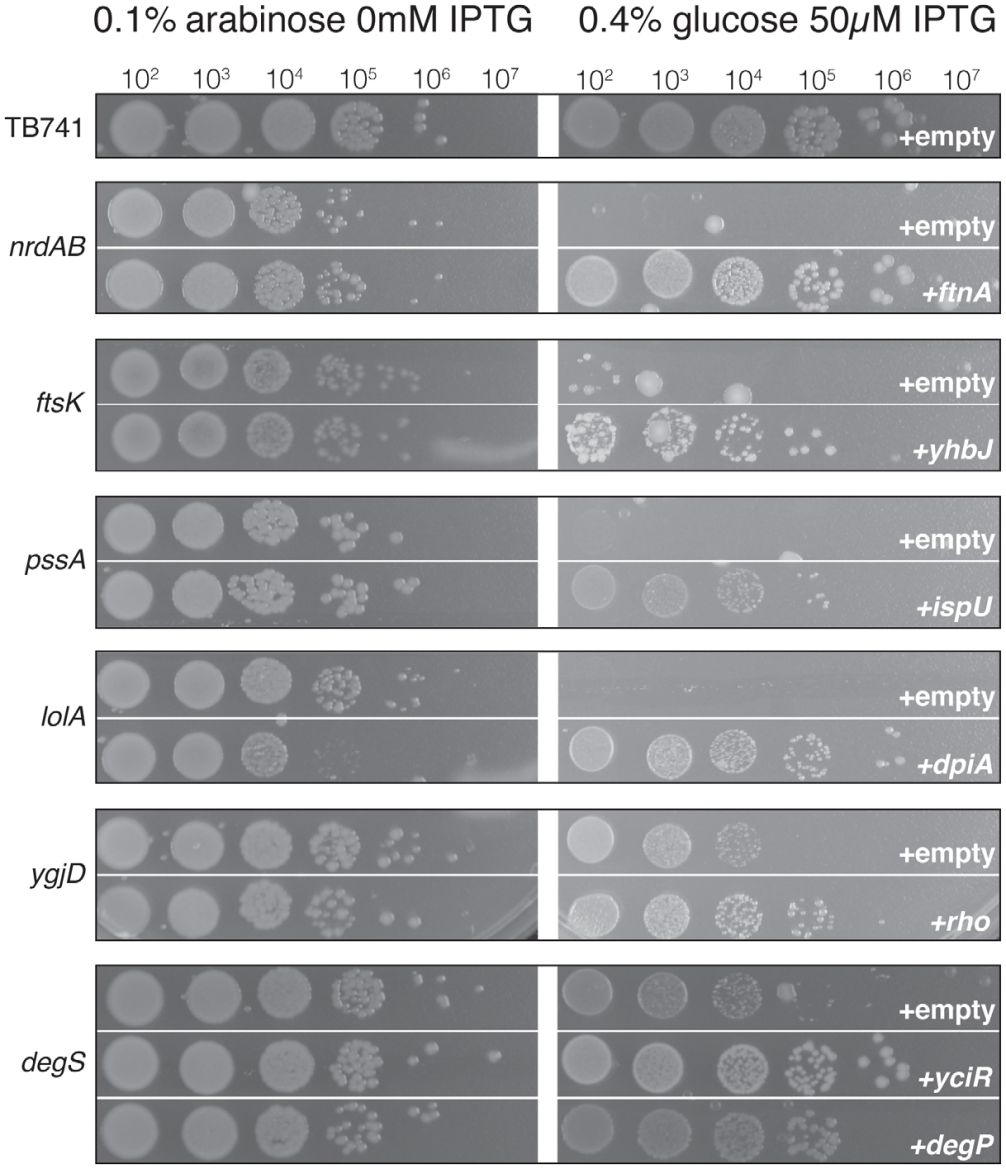
Differential colony formation of conditional lethal mutant strains with suppressive plasmids. In six cases we isolated HCS, but were unable to knockout the corresponding essential gene even in the presence of the HCS. For each essential gene, the top row illustrates the growth that is observed when the native promoter is replaced by P_ara_, under permissive (0.1% arabinose) or restrictive (0.4% glucose) conditions. The bottom row indicates the growth observed for the same strain containing the plasmid with the corresponding HCS, under permissive (0.1% arabinose) or restrictive (0.4% glucose) conditions. Expression of the HCS was induced with 50 µM IPTG. When expression of the essential gene is repressed, robust growth is only observed in the presence of the high copy suppressor. To grow the spot plates, cultures were grown overnight in 0.1% arabinose with 15 µg/ml chloramphenicol, diluted into LB medium, and 5 µl of the indicated dilution were spotted onto plates containing either 0.1% arabinose, or 0.4% glucose with 50 µM IPTG. The arabinose plates were incubated for 24 hours, while the glucose IPTG plates were incubated for 48 hours at 37°, except for P_ara_-*degS*, which was incubated for 14 hours.

The other six essential genes could not be deleted from strains containing the HCS plasmids. This suggested that in the presence of the HCS, low-level expression of the essential genes was sufficient to allow growth. Without the HCS, this residual low-level expression did not allow growth (Figure 4). Alternatively, it is possible that the high copy suppressor increased expression from P_ara_, and thus restored normal levels of the essential proteins. Therefore, we tested whether any of these seven HCS (two HCS were included for degS) increased expression from the P_ara_ promoter. We used a chromosomally encoded P_ara_-*phoA* fusion to monitor expression from P_ara_ under conditions where expression of the HCS is induced. HCS clones overexpressing *yciR*, *yhbJ*, and *ftnA* exhibited slightly lower levels of PhoA-activity compared to controls, while overexpression of *degP*, *rho* and *dpiA* resulted in slightly elevated levels of PhoA-activity (approximately 1.5-fold increase over the control). However, this activity was more than 50-fold below the activity of P_ara_ when induced with 0.1% L-arabinose (Figure S1). This suggested that none of these HCS rescued the conditional lethal phenotype through increasing expression of the essential gene.

To test if the HCS genes caused a general non-specific rescue, we purified the HCS plasmids from the ASKA collection and transformed these plasmids into each conditional lethal mutant. We tested for regrowth in the same way as in the retransformation test described above. With one exception (see below), none of the HCS plasmids restored growth in any other conditional lethal strain except the strain it was recovered from. Therefore we assumed that the observed high copy suppression is due to a specific mechanistic link between the depletion phenotype and the high copy suppressor (Table 1), rather than a consequence of a high copy suppressor-mediated induction of expression from P_ara_.

**Table 1:** Possible functional similarities between non-complementing high copy suppressors and essential genes.

Essential gene	High copy suppressor	Depletion phenotype	Possible functional similarity
*degS* – serine endoprotease	*degP* – serine protease	Sensitivity to outer membrane stress, temperature sensitivity	RseA cleavage and sigma E activation [30]
*degS* – serine endoprotease	*yciR* – cyclic di-GMP phosphodiesterase/modulator of RNase II	Sensitivity to outer membrane stress, temperature sensitivity	csgD repression and silencing of curli expression [57]; reduction of outer membrane protein folding stress analogous to silencing of *ompA* and *opmC* expression [58]; modulation of RNase II activity analogous to HicA [59]
*ftsK* – cell division protein	*yhbJ* – regulator of *glmS* stability	Inhibition of cell division, DNA damage (filamentation) [60]	Modulation of peptidoglycan structure [61] similar to loss of function in *dacA* [62]
*lolA* – periplasmic chaperone	*dpiA* – response regulator	Membrane defects [63]	Signaling of outer membrane stress [64]
*nrdAB* – ribonucleoside phosphate reductase	*ftnA* - ferritin	Inhibition of DNA replication and cell division (filamentation) [65]	Reduction of oxidative damage and ROS-induced cell death by iron sequestration [26]
*pssA* – phosphatidyl serine synthase	*ipsU* – undecaprenyl diphosphate synthase	Filamentation [66]	Structural lipid analogue similar to cardiolipin [67]
*ygjD* – t^6^A transfer-RNA modification enzyme	*rho* – transcriptional termination	Inhibition of translation [68]	Adjustment of transcript levels and binding of free RNA [69]; Restoration of transcriptional attenuation [70]

We derived depletion phenotypes of conditional lethal mutants and the original function of high copy suppressor genes from literature. Proposed modes of action of the high copy suppressor genes are also based on evidence from literature.

The single exception to this pattern was the HCS *ftnA* (coding for ferritin), which rescued *ftsK*- or *nrdAB* depletion. *ftnA* and *nrdB* exhibit structural homology (Table 2), suggesting that this is a specific functional replacement. In the case of *ftsK*, the mechanism of suppression is less clear. One possibility is that the FtnA protein alleviates oxidative stress [26] that results from the loss of FtsK, as a consequence of double strand breaks in chromosomal DNA [27]. However, in both cases, the mechanism does not appear to be moderated through FtnA restoring expression from P_ara_ (Figure S1).

**Table 2:** Sequence and structural homology of essential genes and their high copy suppressors.

Essential gene	HCS	Amino acid alignment e-value	Structural homology p-value (−ln)
dapA*	*nanA*	4.7e−22	31.26
fldA*	*fldB*	1.7e−33	22.49
pyrH*	*cmk*	0.26	1.23
spoT*	*mutT*	0.14	2.28
*degS*	*degP*	2.7e−47	20.99
*degS*	*yciR*	0.11	2.59
*ftsK*	*yhbJ*	0.58	1.59
*lolA*	*dpiA*	0.85	0.91
*nrdA*	*ftnA*	0.071	0.85
*nrdB*	*ftnA*	0.065	7.64
*pssA*	*ispU*	0.95	2.48
*ygjD*	*rho*	0.01	2.30

The Protein Information Resource (http://pir.georgetown.edu/pirwww/search/pairwise.shtml) was used to generate Smith-Waterman amino acid alignments and the corresponding e-values; lower e-values imply higher homology between sequences [28]. MAMMOTH (Matching Molecular Models from Theory) [29] was used to generate pairwise structural alignments; a value above 4.5 (p = 0.01) indicates significant structural homology [29]; if no structure was available, a structure model from http://modbase.compbio.ucsf.edu/modbase-cgi/index.cgi was used. Essential genes that could be knocked out in the presence of their HCS are indicated with an asterisk.

These data thus show that out of the 23 essential genes or operons that we assessed, the functions of four could be completely replaced by non-orthologous genes. In six additional cases, the functions of the essential genes could be almost completely replaced: over-expression of a second gene enabled cellular viability even when the expression of the essential gene was largely abolished. In contrast, without overexpression of this second gene, no growth occurred.

### Dosage Dependence of Knockout Mutant Strains

**Figure 5:**
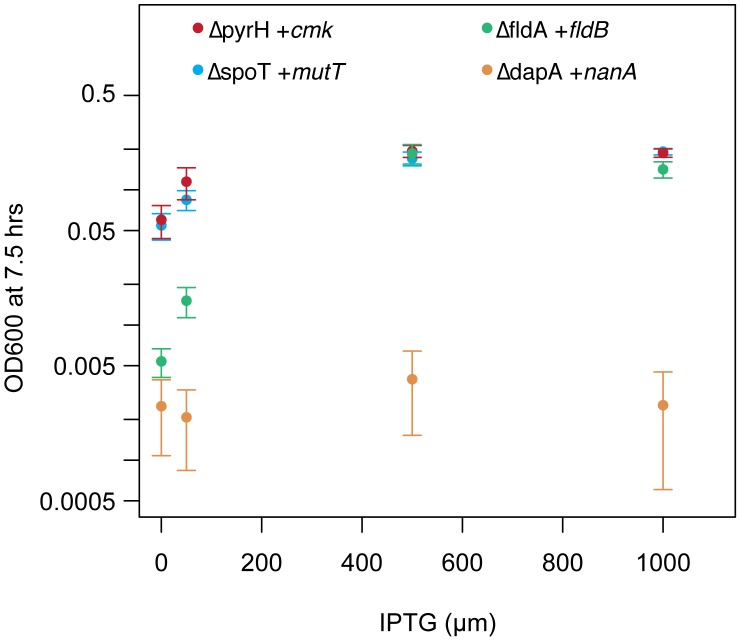
Gene dosage dependence of essential gene knockouts complemented with high copy suppressors. The strains in which we were able to knockout the essential genes were grown in the presence of their respective HCS and the growth yields after 7.5 hours of growth were measured using optical density (OD) at 600 nm. The *dapA* knockout complemented with *nanA* exhibited nearly undetectable growth at all levels, suggesting that this complementation is very weak. All other mutants exhibited significantly increased yields at 50 µM IPTG, and near maximal growth yields at 500 µm IPTG. For *pyrH* and *spoT*, growth in 0 µM IPTG presumably results from leaky expression from P_lac_, or from carryover of gene products or small amounts of IPTG from the overnight growth cultures (see Figure 3 and Methods). Error bars indicate 95% confidence intervals.

We also quantified how well the high copy suppressors restored growth in the four strains in which we could knock out the essential gene, by measuring how the growth yield depended on the dosage of HCS expression. The complemented knockout mutants showed qualitatively different responses to increasing expression of the HCS, as measured by the amount of the inducer IPTG added to the growth medium (Figure 5). The *dapA* knockout exhibits very low levels of growth at all levels of inducer, suggesting that NanA has only a low level of activity toward the DapA substrate, or that very high levels of activity are required to sustain growth. On the other hand, both the *spoT* and *pyrH* knockouts exhibit growth even when the suppressor is uninduced, suggesting that either these proteins are much more promiscuous, or that only low levels of activity are required for growth (Figure 5).

### Amino Acid and Structural Similarities between High Copy Suppressors and Essential Genes

To gain insight into the mechanisms of suppression, we compared the amino acid sequences (using Smith-Waterman alignments [28]) and protein structures (using pairwise structural alignments [29]; Table S2) of all complementing HCS – essential gene pairs. Of the four gene pairs for which we could knock out the essential gene, two HCS share homology in amino acid sequence and protein structure with the suppressed essential gene: *dapA* and *nanA* share amino acid homology (Table 2), with *nanA* being the closest homologue of *dapA* in *E. coli*. However, phylogenetic analysis shows that they are only distantly related, and most likely diverged before the most recent common ancestor of all bacteria (Figure S2B). The second HCS – essential gene pair showing amino acid homology is *fldA* and *fldB* (Table 2), with *fldB* being the closest homologue of *fldA* in the *E. coli* genome. Phylogenetic analysis suggests that these two proteins diverged after the origin of gamma-proteobacteria (Figure S2A).

In the set of six HCS for which we were unable to knock out the corresponding essential gene, one pair exhibits sequence homology: the proteins DegS and DegP [30] (Table 2). Again, *degP* (with *degQ*) is the closest homologue of *degS* in the *E. coli* genome.

### Functional Similarities between HCS and Essential Genes

In several cases, the functions of the essential gene and its complementing HCS appear to be related. Besides having amino acid and structural similarities, the three pairs mentioned above (*dapA*-*nanA*, *fldA*-*fldB*, and *degS*-*degP*) have known functional similarities.

DapA and NanA both belong to the N-acetylneuraminate lyase subfamily and catalyze similar biochemical reactions [31], although *dapA* is essential and *nanA* is non-essential. It has previously been shown that a single amino acid exchange can turn NanA, an N-acetylneuraminate lyase, into an efficient dihydrodipicolinate synthase, which is the dedicated function of DapA. This amino acid exchange was hypothesized to optimize the turnover rate rather than the specificity of the reaction [32]. Therefore, replacement of DapA by NanA may be an example of how the increased concentration of an enzyme with promiscuous activity can promote an essential biochemical reaction and restore viability in the absence of the gene originally encoding the essential function.

FldA and FldB are both flavodoxins. FldB is non-essential, in contrast to FldA [1], and cannot replace the function of FldA when expressed from its native promoter [33]. However, our results show that that FldB can replace the function of FldA when expressed at a high level.

DegS activates the sigma E stress response via proteolytic degradation of the anti-sigma factor RseA [34] triggered by misfolded outer membrane porins [35], and it seems that DegP can fulfill the same function [30]. Although this implies that DegP overexpression might fully compensate for DegS function, we were unable to delete *degS* when overexpressing *degP*.

Two of the essential gene – HCS pairs that exhibit no apparent amino acid or structural similarity do exhibit functional similarity: *pyrH*/*cmk* and *spoT*/*mutT*. c*mk* and *pyrH* both code for a nucleotide kinase: PyrH converts uracil monophosphate to uracil diphosphate, while Cmk converts cytosine monophosphate to cytosine diphosphate. It is also known that Cmk can use both cytidine and uridine (the primary substrate of PyrH) as substrates. Additionally, it has been shown previously that *cmk* can act as a high-copy suppressor of a temperature-sensitive *pyrH* allele [36]. Here we have shown that *cmk* is fully suppressive by deleting the entire *pyrH* locus.

Both *mutT* and *spoT* can recognize phosphorylated guanosines as substrates, and cleave phosphoryl groups. SpoT is a key enzyme of the stringent response and hydrolyzes penta/tetra guanosine phosphate ((p)ppGpp) [37], [38]. Deletion of *spoT* leads to the accumulation of (p)ppGpp, which in turn activates stringent response and leads to cessation of cell growth. One possible mechanism of suppression is that *mutT* can cleave phosphoryl groups from (p)ppGpp, converting it into a phosphorylated guanosine that no longer triggers the stringent response, thus allowing cell growth.

Other possible functional similarities between pairs of high copy suppressors and essential genes are listed in Table 1.

We also objectively evaluated the hypothesis that there are specific functional relationships between essential genes and their HCS by testing whether the functional annotations of essential genes and their HCS are more similar than expected by chance. We collected all the GO molecular function annotations [39] for each essential gene and its corresponding HCS, and calculated a functional distance between each pair (see Methods). Using this functional distance measure, we found that essential genes and their complementary HCS genes are much more similar in function than would be expected by chance (p = 0.0024, one-tailed Kolmogorov-Smirnov test). When we exclude the homologous pairs of genes (*degS*-*degP*, *dapA*-*nanA*, and *fldA*-*fldB*, a weak signal of functional similarity remains (p = 0.05, one-tailed Kolmogorov-Smirnov test). Thus, it appears that the HCS genes compensate for the deleted essential genes through specific complementation of the missing function. On the other hand, *ybiV*, which is likely to be a non-specific HCS, shows no pattern of having greater functional similarity to its paired essential genes than would be expected by chance.

### Correlation with Evolutionary Patterns

Comparative genomic analyses have shown that genes that are essential in *E. coli* tend to be conserved in other bacterial taxa [12]–[15]. In addition, recent empirical results have shown that the essential functions of genes tend to be conserved: genes that are essential in one taxon have orthologues that are essential in other taxa (Figure 1). There are, however, exceptions: some essential genes are less well conserved, or have become non-essential in some taxa.

Here, we have shown that under laboratory conditions, the functions of many essential genes can be completely or partially replaced by homologous or unrelated non-essential genes. A simple explanation might connect these two observations: if it is difficult to replace the function of an essential gene, then this gene should be both highly conserved and consistently essential across bacterial taxa. We thus asked whether it is more difficult to find genes providing compensatory functions for genes that are both conserved and consistently essential.

We used data on conservation (see Methods) and empirical assessments of essentiality [1], [3]–[11] to test this hypothesis. Of the 23 essential genes or operon pairs that we investigated, eight are both conserved and essential for all bacteria in which essentiality has been empirically assessed (Figure 1). Within this set, we found an HCS for only a single gene, *ygjD*. 15 of the essential genes or operons that we considered are either not fully conserved across bacteria or are nonessential in some taxa, or both. Of these, we found HCS for 9 of the 15; if we exclude the operon pairs, as it may generally be more difficult to find compensatory functions for both genes, this fraction increases to 8 out of 12. The probability of finding so few HCS for conserved and consistently essential genes by chance is 0.037 and 0.025, respectively (one-tailed Fisher's exact test). The estimated odds ratios are (with 95% upper limits in parentheses): 0.11 (0.89) and 0.083 (0.75), respectively. These data suggest that suppressors of conserved consistently essential genes are approximately one fifth as likely to be found as suppressors for genes that are less conserved or are non-essential in some taxa.

Thus, genes that are ancient, strongly conserved, and consistently essential across taxa appear to be persistently essential under laboratory conditions.

### Conclusions

The data here support the hypothesis that in some cases, simply increasing the expression level of specific non-essential genes can render essential genes non-essential (i.e. high copy suppression). Sequence and structural comparisons showed that some of the HCS genes were homologous to the essential genes whose function they replaced. However, these homologues tended to be distantly related, with divergence times ranging from before the root of all bacteria, to soon after the origin of gamma-proteobacteria. In addition, in the majority of cases, homology was not required for HCS to occur, highlighting the possibility that even when there is no detectable homology, elevated expression can act as a mechanism allowing functional replacement. For example, although the essential gene – HCS pairs *spoT*-*mutT* and *pyrH*-*cmk* do not share detectable similarity on protein structure or sequence level, their biochemical activity is apparently similar enough to allow complete functional complementation.

In several instances, the HCS that we recovered did not allow the deletion of the corresponding essential gene. We hypothesize that a combination of very low expression levels of the essential gene and expression of the relevant HCS allowed suppression of the lethal phenotype. However, in almost all cases, the suppression of the conditional lethal phenotype seemed to be based on a specific mechanistic link, a hypothesis that was further supported by the finding that the molecular functions of essential genes and their dedicated high-copy suppressors are far more similar than would be expected by chance.

Finally, we have shown that when the function of an essential gene can be replaced in the laboratory, orthologues of that gene are more likely to be non-essential or absent from the genomes of other bacterial taxa. This observation suggests that compensability may influence patterns of evolutionary conservation: the functions of some essential genes are easier to replace than others, and the genes that perform such functions may be lost more often over evolutionary time.

Previous studies have looked for high copy suppressors of lethal phenotypes [25], [40], [41]. The majority of these studies have been performed on a smaller scale or by screening for suppressors of mutations that cause non-lethal phenotypes, with the aim to investigate gene function. Our study is a comparative and systematic attempt to quantify the frequency of suppressors of essential genes, and to test if there is a statistical association between gene conservation and compensability.

The potential for finding redundant, yet non-orthologous genes that can functionally replace essential genes might be a function of genome size. Previous work has shown that bacterial species with large genomes have fewer essential genes than species with small genomes [4], [5]. One explanation for this observation is that in large genomes, there is a greater chance that a second gene encodes a similar function. Thus, the chance to replace essential gene functions with other functions could be greater in species with larger genome sizes and a generalist lifestyle. It would thus be interesting to test how the results of this study compare with additional studies in bacteria having much larger (or smaller) genomes. Indeed, in bacteria with small genomes, almost all essential genes are also highly conserved; thus finding conditionally essential genes may prove far more difficult.

Overall, our work provides a novel explanation for the different patterns of conservation that are observed for essential genes, and emphasizes that gene essentiality is a fluid characteristic, even over short periods of evolutionary time.

## Methods

### Strains and Media

All strains were grown in LB media (Sigma) or LB agar plates (1.5% agar, Sigma), and L-arabinose or D-glucose (both Sigma) was supplemented as indicated. *E. coli* strains MG1655 and DY330 were described previously [42], [43] and grown at 37°C and 32°C, respectively, with vigorous shaking. AB330 is a Lac^+^ derivative of DY330, and was received from Alex Boehm, University of Wurzburg, Germany. P1 transduction and TSS transformation were done as described elsewhere [44], [45]. Strains harboring a pKD4 derived kanamycin resistance cassette were grown with 50 µg/ml kanamycin sulfate (Sigma), and strains with ASKA(-) plasmids with 15 µg/ml chloramphenicol (Calbiochem). Ampicillin (Fluka) 25 µg/ml was used to select for P_ara_-*phoA* insertion in *attB*. Strains transformed with pCP20 [46] were grown at 32° in the presence of 15 µg/ml chloramphenicol (Calbiochem). IPTG (isopropyl thiogalactopyranoside) was from Sigma.

### Assembling a Collection of Conditional Lethal Mutant Strains

No comprehensive collection of conditional lethal mutants of essential genes is available. To construct a collection, we selected 23 essential genes and operon pairs from *E. coli* that exhibited varying levels of conservation across other bacterial taxa [15]. Genes were balanced for functional categories, but otherwise random. This group of essential genes covers nearly 10% of the essential gene content of *E. coli* MG1655.

Before we selected essential genes for our experiment, we discarded genes located in operons coding for other essential genes, because insertion of the P_ara_ construct in front or inside operons might have strong polar effects. Three exceptions were made: *nrdAB*, *dnaTC* and *glmUS* are essential tandem operons whose gene products interact physically or are involved in the same cellular processes. We assumed that the construct we use to repress transcription abolishes expression of both genes.

### Template Strain Construction

We used the previously described strain TB55 [47] as PCR template for construction of arabinose-inducible conditional lethal mutants (analogous to Roux et al. [48]), with the aim of tightly linking a kanamycin marker to the arabinose-inducible promoter of the *araBAD* operon. This strain allows the generation of a PCR product that contains an outward facing kanamycin resistance marker on one end, and on the other end an outward facing arabinose-inducible promoter. Insertion of this construct in front of essential genes and fusion of the P_ara_ promoter to transcriptional or translational start sites allows control of expression of selected essential genes [48]. We used TB55 to generate PCR products flanked by 40 to 42 base pairs homology to the upstream region of essential genes of interest. The PCR product spanned the kanamycin resistance gene, *araC* and the full intergenic region between *araC* and *araB*.

Next, we constructed TB741, a strain that allowed us to monitor expression of P_ara_ from a second, independent arabinose-inducible *araBAD* promoter. To that end, we combined a *phoA* knockout acquired from KEIO clone JW0374 [1], and, after removal of the kanamycin resistance marker with pCP20 [46], a P_ara_-*phoA* construct was inserted into *attB* (derived from *E. coli* strain SA22 (a gift from Prof. Winfried Boos, University of Konstanz, Germany) with P1 phage transduction. All strains used in this study can be found in Table S3.

### Primer Design for Conditional Lethal Mutants

All conditional lethal mutant strains were constructed initially with the same primer design, which included the following: deletion of 40 to 100 base pairs of the upstream region of the gene of interest by insertion of the PCR product generated from TB55, and fusion of the start codon of the gene of interest with the start codon of *araB*.

We were not able to recover clones with a conditional lethal character for *yeaZ* and *murA* following this methodology. Therefore we fused the transcription initiation site of *araB* to the predicted transcriptional start sites of *murA* and *yeaZ* (from www.regulondb.ccg.unam.mx), yielding conditional lethal clones. All oligonucleotide sequences can be found in Tables S4 and S5.

### Promoter Swap

As mentioned above, strain TB55 was used to generate PCR products that contained a kanamycin cassette adjacent to *araC*, the full P_ara_-region and 42 to 45 base pairs at the 5′ and 3′ -prime ends that were homologous to the upstream and N-terminal region of the essential gene of interest. DY330 cells were grown in LB medium supplemented with 0.2% arabinose and made electro- and recombination competent as described previously [49]. After electroporation, cells were rescued in LB medium containing 0.2% arabinose and incubated at 32° for 1.5 hours prior to plating on arabinose- and kanamycin - containing LB plates. Clones were checked on LB plates supplemented with 0.4% glucose to confirm their conditional lethal character. The constructs were then moved by P1-transduction into TB741, and conditional lethality was assessed again on LB plates with 0.4% glucose. All promoter fusions as well as the adjacent *araC* gene were verified by sequencing.

### Construction of the Plasmid Library

We used the ASKA(-) strain collection [50] to construct a plasmid pool that contained all *Escherichia coli* open reading frames. The ASKA(-) library consists of 4123 clones, each one carrying a plasmid with one open reading frame. We pin-replicated clones into 96-well plates containing LB medium (Sigma) and 15 µg/ml chloramphenicol. Plates were incubated for 48 hours at 37°C. Then, 20 µl of each well were pooled, but clones containing plasmids that coded for essential genes of interest in our experiment were excluded. Plasmids were extracted using a plasmid preparation kit (Promega), following the recommendations of the manufacturer.

### Transformation of Conditional Lethal Mutants

Each conditional lethal mutant was grown in LB medium with 0.1% arabinose (Sigma) to an OD_600 nm_ of 0.4 to 0.8. During the preparation of electrocompetent cells, the density of all cultures was adjusted to an OD_600 nm_ of 1 to guarantee an equal number of cells per transformation event. Each clone was electroporated with 1 µl of plasmid pool (DNA concentration approximately 330 ng/µl). Cells were rescued with 1 ml LB medium with 15 µg/ml chloramphenicol, and 100 µl was immediately removed and transferred to 900 µl LB medium with 0.1% arabinose and 15 µg/ml chloramphenicol to estimate transformation efficiency. To select for high copy suppressors, cells were spread on LB agar plates containing 0.4% glucose (to enhance repression of P_ara_), 50 µM IPTG and 15 µg/ml chloramphenicol, and incubated at 37°C until colonies appeared, or maximally 3 days to minimize the formation of colonies that might arise due to chromosomal suppressor mutations.

### Selection and Verification of High Copy Suppressor Plasmids

We recovered up to 10 colonies per transformation event and restreaked them onto plates with glucose, IPTG, and chloramphenicol to verify growth. After successful regrowth, clones were grown in liquid cultured overnight with 0.1% arabinose and plasmid was extracted (Promega). The purified plasmid was retransformed [44] into fresh ancestral conditional lethal mutant strains under permissive conditions, and 4 independent colonies of each transformation event were tested on permissive and restrictive plates for growth. This procedure directly tested for suppression mediated by more than one plasmid: only upon successful regrowth of all 4 clones, were the plasmids sequenced using the primer 5′-GCGGATAACAATTTCACACAGA-3′. Cases in which all four clones did not grow were discarded from further analysis. The outcome of this retransformation test was based on the transformation method that exhibits a comparably low efficiency [44], decreasing the probability of transforming two different plasmids into the same cell, making it unlikely that more than one plasmid was responsible for high copy suppression.

After this verification procedure, we went back to the original ASKA(-) library, purified plasmids that we recovered from the screen (except *yciR*; this gene was not contained in the clone at the indicated position in the collection), and repeated the procedure. This led to exclusion of two high copy suppressors for *aspS*, and verified all other suppressive plasmids we found in the screen.

### PhoA Assay

To determine if expression of a high copy suppressor lead to strongly increased expression of the P_ara_ promoter, we assayed the activity of a P_ara_-*phoA* fusion inserted into the lambda attachment site, using a previously described procedure [51]. Briefly, we transformed plasmids coding for high copy suppressors (and as a control the empty plasmid pCA24N) into TB741, and grew clones overnight with 50 µM IPTG, 0.4% glucose and 15 µg/ml chloramphenicol in 96 well plates, replicating each clone independently 16 times. To estimate the maximum expression level of P_ara_-*phoA*, we induced 16 replicates of TB741 harboring pCA24N with 0.1% arabinose. After overnight growth, cultures were spun down, resuspended in phoA buffer (150 mM TrisHCl adjusted to pH 9), diluted 1∶2 into fresh phoA buffer to a volume of 180 µl, and the OD600 nm was measured. One drop (approximately 10 µl) of a 1% SDS solution (Sigma) and 25 µl of a 10 mg/ml PNPP (4-nitrophenylphosphate, Sigma) solution was added. After incubation at room temperature for 24 hours the OD_550 nm_ and OD_420 nm_ were measured, and PhoA activity determined using the formula described in [51].

### Knockout of Essential Genes

To delete *spoT*, *pyrH*, *fldA* and *dapA*, plasmids encoding *mutT*, *cmk*, *fldB* and *nanA* were transformed into AB330 and expression was induced with 50 µM of IPTG (or 1 mM for *nanA*). Knockouts were achieved following previously described methods [43], [49] using a pKD4-derived kanamycin cassette flanked by homologous ends. Successful deletions were moved into MG1655 (harboring ASKA(-) plasmids coding for HCS) with P1 transduction [45] with addition of IPTG and verified by PCR using primers upstream and downstream of the insertion.

### Cross-Transformation Tests

To test for specific interactions between the depletion of essential genes and expression of plasmid-based non-complementing high copy suppressors, the HCS plasmids purified from the ASKA(-) library were transformed under permissive conditions into each conditional lethal mutant, and regrowth was checked as described for the initial screening procedure.

### Dosage Response Measurements

Gene deletion mutants were grown overnight at 37°C in 96-well plates with shaking at 400 rpm in 8-fold replication, in LB medium supplemented with 1 mM IPTG and 15 µg/ml chloramphenicol. Cultures were spun down, washed once in LB medium, and diluted 1∶10^−4^ into fresh medium with IPTG concentrations as indicated. Optical density at 600 nm was measured every 30 minutes, for 7.5 hours in total.

### Differential Growth of Conditional Lethal Mutant Strains and Plasmid-Complemented Mutants

To analyze differential growth of conditional lethal mutant strains (with suppressive plasmids, empty control plasmids, deletion of essential genes or ancestral conditional lethal mutants), we grew the corresponding clones in 96-well plates overnight. As a control, each conditional lethal mutant and the ancestral TB741 strain were transformed with the empty plasmid pCA24N. Conditional lethal mutants were grown with 0.1% L-arabinose and, if required, in presence of 15 µg/ml chloramphenicol to select for ASKA(-) plasmids. Essential gene deletion mutants were cultured with 1 mM IPTG to induce expression of high copy suppressors and to decrease the likelihood of genetic suppressor mutations. After overnight growth, cultures were serially diluted by repeatedly transferring 20 µl of culture into 180 µl of LB medium. Of this dilution series, 5 µl of the indicated dilutions were spotted onto plates supplemented with arabinose, glucose, chloramphenicol and IPTG as indicated and incubated as indicated.

### Assignment of Orthology

We used assignments based on previously published data [15]. Briefly, we used reciprocal shortest distance [52] to find potential orthologues of the relevant *E. coli* genes in the respective genomes. Two genes that are reciprocally the most closely related were denoted as orthologues if they aligned over more than 60% of the longer gene. In cases in which no orthologues were found, we used the MicrobesOnline database to search for genes named as putative orthologues. In this way, we found two additional putative orthologues, one for *ftsK* in *S. pneumonia*, and a second for *plsC* in *S. aureus*.

We used data from ten empirical studies on essentiality [1], [3]–[11] to determine whether or not genes orthologous to those in *E. coli* were essential in other bacterial taxa.

### Phylogenetic Analyses

Orthologous and homologous genes from a range of bacterial taxa were selected and aligned using Muscle v3.8.31 [53] with default parameters. The alignments were cleaned using GBlocks 0.91b [54] with length of non-conserved positions set to 32, the number of flank and conserved positions set to minimum values, minimum block length to 2, and allowed gaps set to all. This alignment was used as input into MrBayes 3.1.2 [55] with a mixed amino acid model and invariant plus gamma distributed rate variation across sites. The chains were run for 200,000 (*dapA*/*nanA*), or 1,000,000 (*fldA*/*fldB*) generations, and the last 20% of the run was used for construction of a majority rule tree.

### Assessment of Functional Similarity

We obtained molecular function annotations from the GO database (www.geneontology.org/GO.downloads.annotations.shtml; 5/20/2011) for all annotated E. coli genes. We also obtained the relationships between all GO categories (www.geneontology.org/GO.downloads.ontology.shtml; OBO v1.2). GO annotations are related in a tree-like manner, beginning with broad, non-specific parent categories (e.g. “binding”), each of which have more specific child categories (e.g. “acyl binding”). Thus, we quantified functional distance as the number of parent categories that separate any two genes, normalized by the total number of parent categories for each gene. We calculated this distance between each essential gene and all other genes in the genome, and compared this to the distance between the essential genes and their complimentary HCS. This yields a number between 0 and 1, specifying the fraction of genes in the genome that are less functionally similar than the essential gene and its HCS. If functional similarity does not play a role for the essential gene - HCS pairs, we would expect this number to be 0.5, on average, and distributed uniformly between 0 and 1. Instead, we found that for 12 out of 13 essential gene HCS pairs, this distance was less than 0.5 (i.e. they were more similar than the average pair of genes); for 8 out of 13 pairs, the distance was less than 0.25. We used a Kolmogorov-Smirnov test to compare this distribution to the distribution expected if there were no functional relation between the essential gene and its HCS (the uniform distribution).

### Statistical Methods

All statistical tests were done in R v2.13.1 [56].

## Supporting Information

Figure S1 Expression of P_ara_ measured as phoA activity. Strain TB741, with a phoA reporter fused to the P_ara_ promoter, was transformed with the indicated plasmids and grown overnight with 50 µM IPTG and 0.4% glucose or 0.1% arabinose. None of the expressed HCS led to *phoA* expression comparable to induction with 0.1% arabinose, although some differences between HCS are apparent. Each strain was replicated 16-fold; Error bars indicate one standard error.
(PDF)

Figure S2 Phylogenetic relationships of homologous essential gene – HCS pairs. A. *fldA* and *fldB* duplicated after the origin of gamma-proteobacteria; outside of gamma-proteobacteria, the genes are present in only a single copy that is presumably orthologous to *fldA*. B. *dapA* and *nanA* are anciently duplicated genes, with both genes being present in bacterial clades that diverged soon after the root of all bacteria (e.g. Bacilli and proteobacteria). Circles at the nodes of the trees indicate the posterior probability support for each node; black: greater than 0.99; white: between 0.5 and 0.9.
(PDF)

Table S1 Transformation efficiencies and complementing plasmids. All genes except *fldA* and *murA* (bolded) were transformed with greater than 1.2×10^4^ high copy suppressor plasmids, which is the number required to cover 95% of the genes in *E. coli*. No complementing plasmids were found for *nrdAB* and *dnaTC*, presumably because no single gene was capable of replacing the function of two genes.
(DOC)

Table S2 PDB input chains for pairwise structural alignments. The PDB names of the essential genes and their complementing high copy suppressors are indicated.
(DOC)

Table S3 Strains used in this study.
(DOC)

Table S4 Oligonucleotides used to construct the strains with conditional expression of essential genes.
(DOC)

Table S5 Oligonucleotides used to construct the deletion strains.
(DOC)
