# Supplementary figures and images for: Patterns of Evolutionary Conservation of Essential Genes Correlate with Their Compensability
Source: PLoS Genet. 2012 Jun 28;8(6):e1002803. doi: 10.1371/journal.pgen.1002803 (PMC3386227; doi:10.1371/journal.pgen.1002803)

PhoA activity

1700

1600

1500

100

80

60

40

20

0

TB741 expressing

*dpiA*

*rho*

*ispU*

*degP*

*yciR*

*yhbJ*

*ftnA*

pCA24N

pCA24N  
0.1% ara

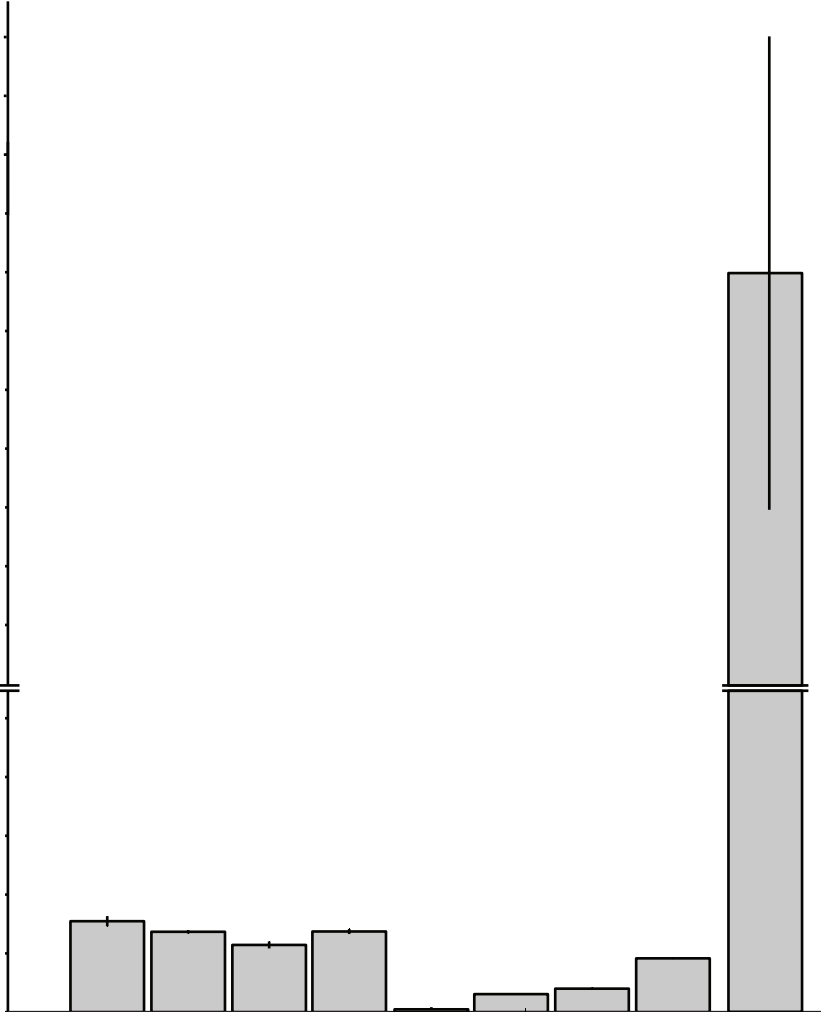

Supplement: Figure S1 — Expression of Para measured as phoA activity. Strain TB741, with a phoA reporter fused to the Para promoter, was transformed with the indicated plasmids and grown overnight with 50 µM IPTG and 0.4% glucose or 0.1% arabinose. None of the expressed HCS led to phoA expression comparable to induction with 0.1% arabinose, although some differences between HCS are apparent. Each strain was replicated 16-fold; Error bars indicate one standard error. (PDF) [file pgen.1002803.s001.pdf]
