# Supplementary material for: Patterns of Evolutionary Conservation of Essential Genes Correlate with Their Compensability
Source: PLoS Genet. 2012 Jun 28;8(6):e1002803. doi: 10.1371/journal.pgen.1002803 (PMC3386227; doi:10.1371/journal.pgen.1002803)
Supplement: Figure S2 — Phylogenetic relationships of homologous essential gene – HCS pairs. A. fldA and fldB duplicated after the origin of gamma-proteobacteria; outside of gamma-proteobacteria, the genes are present in only a single copy that is presumably orthologous to fldA. B. dapA and nanA are anciently duplicated genes, with both genes being present in bacterial clades that diverged soon after the root of all bacteria (e.g. Bacilli and proteobacteria). Circles at the nodes of the trees indicate the posterior probability support for each node; black: greater than 0.99; white: between 0.5 and 0.9. (PDF) [file pgen.1002803.s002.pdf]

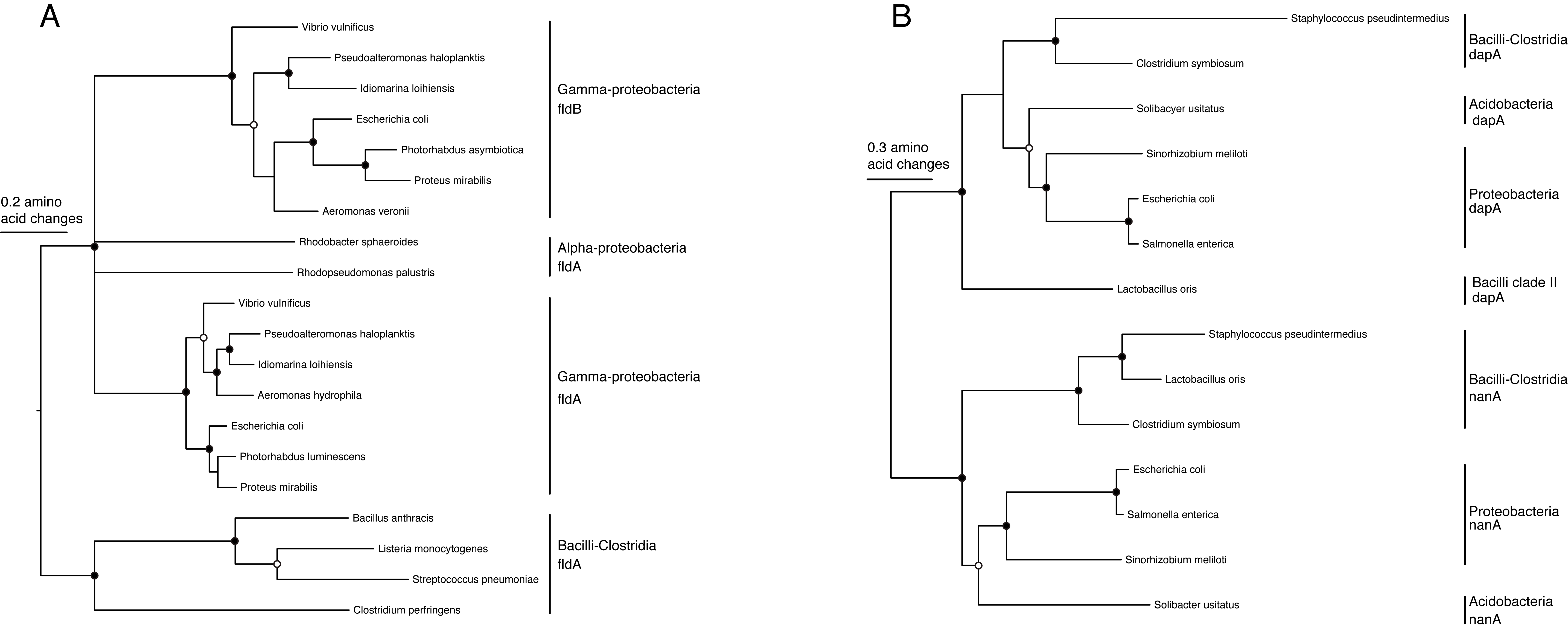
A
0.2 amino acid changes
Vibrio vulnificus
Pseudoalteromonas haloplanktis
Idiomarina loihiensis
Escherichia coli
Photorhabdus asymbiotica
Proteus mirabilis
Aeromonas veronii
Gamma-proteobacteria fldB
Rhodobacter sphaeroides
Rhodopseudomonas palustris
Alpha-proteobacteria fldA
Vibrio vulnificus
Pseudoalteromonas haloplanktis
Idiomarina loihiensis
Aeromonas hydrophila
Escherichia coli
Photorhabdus luminescens
Proteus mirabilis
Gamma-proteobacteria fldA
Bacillus anthracis
Listeria monocytogenes
Streptococcus pneumoniae
Clostridium perfringens
Bacilli-Clostridia fldA
B
0.3 amino acid changes
Staphylococcus pseudintermedius
Clostridium symbiosum
Bacilli-Clostridia dapA
Solibacyer usitatus
Acidobacteria dapA
Sinorhizobium meliloti
Escherichia coli
Salmonella enterica
Proteobacteria dapA
Lactobacillus oris
Bacilli clade II dapA
Staphylococcus pseudintermedius
Lactobacillus oris
Clostridium symbiosum
Bacilli-Clostridia nanA
Escherichia coli
Salmonella enterica
Sinorhizobium meliloti
Proteobacteria nanA
Solibacter usitatus
Acidobacteria nanA
