# Supplementary material for: Patterns of Evolutionary Conservation of Essential Genes Correlate with Their Compensability
Source: PLoS Genet. 2012 Jun 28;8(6):e1002803. doi: 10.1371/journal.pgen.1002803 (PMC3386227; doi:10.1371/journal.pgen.1002803)
Supplement: Table S1 — Transformation efficiencies and complementing plasmids. All genes except fldA and murA (bolded) were transformed with greater than 1.2×10^4^ high copy suppressor plasmids, which is the number required to cover 95% of the genes in E. coli. No complementing plasmids were found for nrdAB and dnaTC, presumably because no single gene was capable of replacing the function of two genes. (DOC) [file pgen.1002803.s003.doc]

| Essential gene | Complementation by ASKA(-) plasmid | Transformants |
| --- | --- | --- |
| *adk* | JW0463 | 1.64 e6 |
| *dapA* | JW2463 | 2.07 e6 |
| *degS* | JW3204 | 1.22 e6 |
| *dnaTC* | - | 1.90 e5 |
| *ffh* | JW5414 | 8.00 e5 |
| ***fldA*** | JW0671 | 1.26 e3 |
| *ftsK* | JW0873 | 4.08 e6 |
| *glmUS* | JW3708 (*glmU*) | 3.36 e6 |
| *gltX* | JW2395 | 2.95 e6 |
| *gyrA* | JW2225 | 2.20 e5 |
| *lolA* | JW0874 | 1.30 e6 |
| *metK* | JW2909 | 6.90 e5 |
| ***murA*** | JW3156 | 7.20 e3 |
| *nrdAB* | - | 3.53 e6 |
| *plsB* | JW4001 | 4.16 e5 |
| *plsC* | JW2986 | 2.18 e6 |
| *proS* | JW0190 | 2.04 e6 |
| *pssA* | JW2569 | 2.32 e6 |
| *pyrH* | JW0166 | 6.37 e5 |
| *spoT* | JW3625 | 9.07 e5 |
| *yeaZ* | JW1796 | 5.30 e5 |
| *ygjD* | JW3036 | 1.94 e5 |
