# Supplementary material for: Patterns of Evolutionary Conservation of Essential Genes Correlate with Their Compensability
Source: PLoS Genet. 2012 Jun 28;8(6):e1002803. doi: 10.1371/journal.pgen.1002803 (PMC3386227; doi:10.1371/journal.pgen.1002803)
Supplement: Table S2 — PDB input chains for pairwise structural alignments. The PDB names of the essential genes and their complementing high copy suppressors are indicated. (DOC) [file pgen.1002803.s004.doc]

| Essential gene | Input chain | High copy suppressor | Input chain |
| --- | --- | --- | --- |
| *fldA* | 1ag9 | *fldB* | 3esz |
| *dapA* | 1dhp | *nanA* | 1fdy |
| *pyrH* | 2bne | *cmk* | 1kdo |
| *degS* | 1soz | *degP* | 1ky9 |
| *degS* | 1soz | *yciR* | 2bas |
| *ygjD* | 2ivn | *rho* | 3ice |
| *ftsK* | 2ius | *yhbJ* | 1kdo |
| *nrdA* | 1r1r | *ftnA* | 1eum |
| *nrdB* | 1mxr | *ftnA* | 1eum |
| *lolA* | 1iwl | *dpiA* | 1A04 |
| *pssA* | 3hsi | *ispU* | 2e98 |
| *spoT* | 1vj7 | *mutT* | 1mut |
