# Supplementary material for: Patterns of Evolutionary Conservation of Essential Genes Correlate with Their Compensability
Source: PLoS Genet. 2012 Jun 28;8(6):e1002803. doi: 10.1371/journal.pgen.1002803 (PMC3386227; doi:10.1371/journal.pgen.1002803)
Supplement: Table S3 — Strains used in this study. (DOC) [file pgen.1002803.s005.doc]

| Name | Relevant genotype | Reference |
| --- | --- | --- |
| MG1655 | *F- lambda-* *ilvG*- *rfb*-50 *rph-1* | Blatter 1997 Science |
| DY330 | lacU169 gal490 cI857 (cro-bioA) | Yu PNAS 2000 |
| AB330 | Lac+ gal490 cI857 (cro-bioA) | Alex Boehm, Wurzburg, Germany |
| TB55 | araC-kan-yabI | Bergmiller BMC Microbiology 2011 |
| TB741 | ∆*phoA*::*frt* attB-D*(latt-lom)::bla* P_ara_-*phoA* | This study |
| CL54 | TB741 Para-*fstK* | This study |
| CL121 | TB741 Para-*nrdAB* | This study |
| CL2101 | TB741 Para-*gyrA* | This study |
| CL7121 | TB741 Para-*spoT* | This study |
| CL31 | TB741 Para-*degS* | This study |
| CL41 | TB741 Para-*plsB* | This study |
| CL61 | TB741 Para-*ffh* | This study |
| CL8 | TB741Para-*dnaT* | This study |
| CL91 | TB741 Para-*gltX* | This study |
| CL10-1 | TB741 Para-*pyrH* | This study |
| CL11-1 | TB741 Para-*lolA* | This study |
| CL13-1 | TB741Para-*adk* | This study |
| CL14-1 | TB741 Para-*proS* | This study |
| CL15-1 | TB741 Para-*metK* | This study |
| CL16-1 | TB741 Para-*plsC* | This study |
| CL17-1 | TB741 Para-*ygjD* | This study |
| CL18-1 | TB741 Para-*fldA* | This study |
| CL19-1 | TB741 Para-*pssA* | This study |
| CL20-1 | TB741 Para-*aspS* | This study |
| CL23-1 | TB741 Para-*glmUS* | This study |
| CL24-1 | TB741 Para-*dapA* | This study |
| CL25-1 | TB741 Para-*murA* | This study |
| CL26 | TB741 Para-*yeaZ* | This study |
| TB103 | ∆*spoT*::kan JW0097(*mutT*) | This study |
| TB104 | ∆*fldA*::kan JW2863(*fldB*) | This study |
| TB105 | ∆*pyrH*::kan JW0893(*cmk*) | This study |
| TB106 | ∆*dapA*::kan JW3194(*nanA)* | This study |
