# Supplementary material for: Patterns of Evolutionary Conservation of Essential Genes Correlate with Their Compensability
Source: PLoS Genet. 2012 Jun 28;8(6):e1002803. doi: 10.1371/journal.pgen.1002803 (PMC3386227; doi:10.1371/journal.pgen.1002803)
Supplement: Table S4 — Oligonucleotides used to construct the strains with conditional expression of essential genes. (DOC) [file pgen.1002803.s006.doc]

| 5_degS_insert | ATGTCCGGTTAACTCGTGGTATGCTGCTGCCGTTCCCTTTAATGTGCCTGTCAAATGGACG |
| --- | --- |
| 3_degS_insert | TTAATCCAATCGCAACGGAACGTAAGAGCTTCACAAACATCGTTTCACTCCATCCAAAAAA |
| 3_degS_verify | AGCTGGTTGTGAGAGTTGGT |
| 5_ftsK_insert | ACACTCCTGTTAATCCATACAGCAACAGTACTGGGGTAACAATGTGCCTGTCAAATGGACG |
| 3_ftsK_insert | TCAATGTGACTTCTTTGTCTTCAATGTATTCCTGGCTCAACGTTTCACTCCATCCAAAAAA |
| 3_tfsK_verify | GGCATCCCACCTAAATTATGG |
| 5_nrdA_insert | AGCTTCCCGTACTACAGGTAGTCTGCATGAAACTATTGCGAATGTGCCTGTCAAATGGACG |
| 3_nrdA_insert | TGCTACCGTCGCGCTTTGTCACCAGCAGATTCTGATTCATCGTTTCACTCCATCCAAAAAA |
| 3_nrdA_verify | GCGGCAGCCTTGATAATGGT |
| 5_plsC_insert | TTTTAACAATGCCCTAACCTGATTTCAGGTGACGTACAATAATGTGCCTGTCAAATGGACG |
| 3_plsC_insert | TGTAAATCACGGTAATAATAAGACGAAAGATATATAGCATCGTTTCACTCCATCCAAAAAA |
| 3_plsC_verify | GCTGAAAGCTACGGCAATGC |
| 5_adk_insert | GGTGCCTTTCTTGAGGCAATCGCCTGTTGGTGGTATCGTTAATGTGCCTGTCAAATGGACG |
| 3_adk_insert | TTTCCCCGCGCCCGGAGCGCCAAGCAGAATGATACGCATCGTTTCACTCCATCCAAAAAA |
| 3_adk_verify | ATGCGCTCTTTAACCAGCGC |
| 5_gyrA_insert | ATAGGTTTACCTCAAACTGCGCGGCTGTGTTATAATTTGCAATGTGCCTGTCAAATGGACG |
| 3_gyrA_insert | CAATGTTGACCGGTGTAATTTCTCTCGCAAGGTCGCTCATCGTTTCACTCCATCCAAAAAA |
| 3_gyrA_verify | TTACGTCACCAACGACACGG |
| 5_spoT_insert | CGCTATTGCTGAAGGTCGTCGTTAATCACAAAGCGGGTCGAATGTGCCTGTCAAATGGACG |
| 3_spoT_insert | AGGTTTGAATCAGTTGATTCAGGCTTTCAAACAGATACAACGTTTCACTCCATCCAAAAAA |
| 3_spoT_verify | AATCACGTCATGCAGCAGCG |
| 5_ygjD_insert | AGTTTTACATCAACCCGCATTGGTCCTACACTGCGCGGTAATAATGTGCCTGTCAAATGGACG |
| 3_ygjD_insert | GCCGGTTTCATCGCAGGAAGTTTCAATACCCAGTACACGCATCGTTTCACTCCATCCAAAAAA |
| 3_ygjD_verify | GGCAATTGGTCTGGGGAGCA |
| 5_dnaT_insert | TCCGTGTGTTACTATAAAAGTTATCTCCCTTCTCGTTCATCGAATGTGCCTGTCAAATGGACG |
| 3_dnaT_insert | GTCAATACCAACGACGTCCGGGGTCAAAACTCTGGAAGACATCGTTTCACTCCATCCAAAAAA |
| 3_dnaT_verify | AGAGTTGATCGTCCAGAGCG |
| 5_fldA_insert | GTCAGGGCATCGTTTAAATTTCCACTTTCATGTAGCACAGAATGTGCCTGTCAAATGGACG |
| 3_fldA_insert | TGAAACTTATTGAAATGGGGGTGGAAAATTGCCCACGGATCGTTTCACTCCATCCAAAAAA |
| 3_fldA_verify | CGAGAGTCGGGAAGAAGTCA |
| 5_lolA_insert | TAGCCTGGAATAGAGAGTAGAGGGAACTCCCGATCGGGAGTGAATGTGCCTGTCAAATGGACG |
| 3_lolA_insert | TAAGCTTGAGAGTAATGCACAGGTGATGGCAATTTTTTTCATCGTTTCACTCCATCCAAAAAA |
| 3_lolA_verify | CAGGTTGTGTCATATGCCAG |
| 5_plsB_insert | CCATGACTTTCTGCTATCCTTGCCGCGCATTTGCATTATTAAAATGTGCCTGTCAAATGGACG |
| 3_plsB_insert | TAAATTCAGTAATTTGTAGTAAATTCGTGGCCAGCCGGACATCGTTTCACTCCATCCAAAAAA |
| 3_plsB_verify | TTTCCAGCGGCTCTAACGGG |
| 5_pssA_insert | CCGCTTATGTCGTCTGCATTGCACAGAGGACCCTTCAATGAAAATGTGCCTGTCAAATGGACG |
| 3_pssA_insert | AAGGTGTTGTTGATGTTTATTACGCTTAAATTTTGACAACATCGTTTCACTCCATCCAAAAAA |
| 3_pssA_verify | GCCTCATACAACGCGTTCAGA |
| 5_yeaZ_insert | GCCCGCGCCACGCACCCGTGACATTGCCCGTGCGGTTCGTAATGTGCCTGTCAAATGGACG |
| 3_yeaZ_insert | TCGCATGGGTCTTCGTGCTTAGATCAATAAAAAGGCGCGCTATGGAGAAACAGTAGAGAGT |
| 3_yeaZ_verify | CAATGCGCACGCCAGTAAAG |
| 5_aspS_insert | GTTGTGACATACAGCTAACGCTGCGACTTGGTCACCTGCGCAAATGTGCCTGTCAAATGGACG |
| 3_aspS_insert | CACGTGGGACAAACGGAGCTGTCCACAATATTCTGTACGCATCGTTTCACTCCATCCAAAAAA |
| 3_aspS_verify | TGCCCGTGACCTGAATGCAG |
| 5_dapA_insert | GCTTGCTTTTAATGCCATACCAAACGTACCATTGAGACACTTAATGTGCCTGTCAAATGGACG |
| 3_dapA_insert | ATCCATCGGAGTAACAATCGCGACAATACTTCCCGTGAACATCGTTTCACTCCATCCAAAAAA |
| 3_dapA_veiry | TTACCGGAATGCGCCCATCA |
| 5_ffh_insert | GACGCCTTCATGTTATACTGCGGCAAAATACTGATGATGTGTAATGTGCCTGTCAAATGGACG |
| 3_ffh_insert | GCGCAGCGTGCGCGACAAACGATCGGTTAAATTATCAAACATCGTTTCACTCCATCCAAAAAA |
| 3_ffh_verify | ATTTTGACGAACTCCTGCCC |
| 5_glmU_insert | CCACCTGACGCTTAAATTAAGGTACTGCCTTAATTTTCTGAATGTGCCTGTCAAATGGACG |
| 3_glmU_insert | ATAGCATTATTCAACATACGCGTCCTGACTGTAATTTGAGCGTTTCACTCCATCCAAAAAA |
| 3_glmU_verify | CTGAACCATCGCTTTCCCGG |
| 5_gltX_insert | GCCCACTTAATTTTTCCAGGATTTGCCGGTTGTCGGCATCTTAATGTGCCTGTCAAATGGACG |
| 3_gltX_insert | ATAGCCTGTTGGGCTTGGCGCGAAGCGAGTTTTGATTTTCATCGTTTCACTCCATCCAAAAAA |
| 3_gltX_verify | AGTACGGACCTTCATCCCAC |
| 5_metK_insert | CAGAGTCGTGGTAGGATCCGCTACCACAGAAAATCCACACAAAATGTGCCTGTCAAATGGACG |
| 3_metK_insert | CCCTTCAGAGACGGACTCGGACGTAAAAAGGTGTTTTGCCATCGTTTCACTCCATCCAAAAAA |
| 3_metK_verify | GAACGGTGTTACGGGTGATC |
| 5_murA_insert | CCGCATTCATGCTGTGTCGATCAAAGCGTATACCCCTGCGAATGTGCCTGTCAAATGGACG |
| 3_murA_insert | CTCAGTTAACAATTCATATCCGCTACCGGCGAATCGCCCATATGGAGAAACAGTAGAGAGT |
| 3_murA_verify | CGCGGGCATCAATATGCACA |
| 5_proS_insert | TTGCACTGGTAAACTAAATCACTTTTTTTTGTCCCAGGCTCGAATGTGCCTGTCAAATGGACG |
| 3_proS_insert | TGTCTCCTTGAGAGTGGAGAGCAGGTATTGGCTAGTACGCATCGTTTCACTCCATCCAAAAAA |
| 3_proS_verify | AAATCGGCTGGCTGAACCAC |
| 5_pyrH_insert | CTTGTGGGGCTGCGCTGAAAAGCGACGTACAATGTCGCTAGTAATGTGCCTGTCAAATGGACG |
| 3_pyrH_insert | AAGCAGAATGCGTTTATAGACGGGTTTTGCATTGGTAGCCATCGTTTCACTCCATCCAAAAAA |
| 3_pyrH_verify | CGCGGTTCATACCCGCTTTC |
| 5_unique | GCTACTCCGTCAAGCCGTCA |
