# Supplementary material for: Patterns of Evolutionary Conservation of Essential Genes Correlate with Their Compensability
Source: PLoS Genet. 2012 Jun 28;8(6):e1002803. doi: 10.1371/journal.pgen.1002803 (PMC3386227; doi:10.1371/journal.pgen.1002803)
Supplement: Table S5 — Oligonucleotides used to construct the deletion strains. (DOC) [file pgen.1002803.s007.doc]

| 5_dapA_KO | TTCTGTCTGCTTGCTTTTAATGCCATACCAAACGTACCATGTGTAGGCTGGAGCTGCTTCG |
| --- | --- |
| 3_dapA_KO | TTTGAACAGAGTAAGCCATCAAATCTCCCTAAACTTTACACATATGAATATCCTCCTTAG |
| 5_dapA_ext | TGACAGTGTCAAACTGGTTA |
| 3_dapA_ext | ACCTGACGCTTATAGCGTGA |
| 5_fldA_KO | TCAATAAGTTTCAAGAGGTTATTTCACTCATGGCTATCACTGGTGTAGGCTGGAGCTGCTTCG |
| 3_fldA_KO | GCGTAGACATGAGTCTACGCCGCATCACATCAGGCATTGAGACATATGAATATCCTCCTTAG |
| 5_fldA_ext | GTGTGCAGTCCTGCTCGTTT |
| 3_fldA_ext | CGGGACATTGTACAGGTACA |
| 5_pyrH_KO | TCGTTGACAGTCTCAGGAAAGAAACATGGCTACCAATGCAAAGTGTAGGCTGGAGCTGCTTCG |
| 3_pyrH_KO | CTTACCCTTATTTATCCATCACGGGAATTATTCCGTGATTAACATATGAATATCCTCCTTAG |
| 5_pyrH_int | AAGTTGAGTGGCGAAGCTCT |
| 3_pyrH_int | CATTGCCAGGCCGTTCATTA |
| 5_pyrH_ext | ATTCAGCTAACCCTTGTGGG |
| 3_pyrH_ext | GACCAAACTGCCTGCAACAA |
| 5_spoT_KO | ACCGCTATTGCTGAAGGTCGTCGTTAATCACAAAGCGGGTCGCCCGTGTAGGCTGGAGCTGCTTCG |
| 3_spoT_KO | GAGCATTTCGCAGATGCGTGCATAACGTGTTGGGTTCATAAAACACATATGAATATCCTCCTTAG |
| 5_spoT_int | TGATTCAAACCTACCTGCCG |
| 3_spoT_int | GGCCTGCGCCTCTTTCTTAT |
| 5_spoT_ext | CTGGTACCGGAAGAAAACGA |
| 3_spoT_ext | TATGAGGTTTGTGGACCTGC |
